# Modelling biochemical networks with intrinsic time delays: a hybrid semi-parametric approach

**DOI:** 10.1186/1752-0509-4-131

**Published:** 2010-09-23

**Authors:** Moritz von Stosch, Joana Peres, Sebastião Feyo de Azevedo, Rui Oliveira

**Affiliations:** 1 LEPAE, Departamento de Engenharia Química, Faculdade de Engenharia, Universidade do Porto, Rua Dr. Roberto Frias s/n, 4200-465 Porto, Portugal; 2 REQUIMTE, Departamento de Quimica, Faculdade de Ciências e Tecnologia, Universidade Nova de Lisboa, 2829-516 Caparica, Portugal

## Abstract

**Background:**

This paper presents a method for modelling dynamical biochemical networks with intrinsic time delays. Since the fundamental mechanisms leading to such delays are many times unknown, non conventional modelling approaches become necessary. Herein, a hybrid semi-parametric identification methodology is proposed in which discrete time series are incorporated into fundamental material balance models. This integration results in hybrid delay differential equations which can be applied to identify unknown cellular dynamics.

**Results:**

The proposed hybrid modelling methodology was evaluated using two case studies. The first of these deals with dynamic modelling of transcriptional factor A in mammalian cells. The protein transport from the cytosol to the nucleus introduced a delay that was accounted for by discrete time series formulation. The second case study focused on a simple network with distributed time delays that demonstrated that the discrete time delay formalism has broad applicability to both discrete and distributed delay problems.

**Conclusions:**

Significantly better prediction qualities of the novel hybrid model were obtained when compared to dynamical structures without time delays, being the more distinctive the more significant the underlying system delay is. The identification of the system delays by studies of different discrete modelling delays was enabled by the proposed structure. Further, it was shown that the hybrid discrete delay methodology is not limited to discrete delay systems. The proposed method is a powerful tool to identify time delays in ill-defined biochemical networks.

## Background

Time delays play a very important role in genetic regulatory systems. Gene regulation and signal transduction as a whole involves the synthesis and maturation of complex proteins. Their synthesis and transport takes a considerable amount of time, which introduces delays in the overall regulation chain. At a process level, metabolic time delays can be observed macroscopically by recognizing a certain time delay between substrate uptake and the corresponding biomass growth or product formation as in cultivations of *Saccharomyces cerevisiae *[1] or *Pichia pastoris *[2]. The nature of time delays in regulatory networks is twofold [3]. They are either related to a process that takes an intrinsic time to be accomplished, i.e. some reactions, such as translational or transcriptional reactions, take a significant amount of time to be completed, or as a consequence of the modelling approach used, i.e. lumping a sequence of events might lead to an apparent time delay.

The bottom-up systems biology approach for building dynamic network models can be too cumbersome due to their complex nature and lack of fundamental knowledge [4-7]. Typical limitations are the involvement of large scale kinetic models with poorly defined kinetic parameters, limited generalization capacity and their cost expansive development. In this paper we propose the use of mathematical hybrid semi-parametric systems as a cost effective alternative to model biochemical networks with intrinsic time delays, since it is not likely to know in advance which fundamental mechanisms cause such delays. Hybrid semi-parametric systems combine fundamental (parametric) biological constraints with more empirical data-based (nonparametric) constraints. Mechanistic and nonparametric models can therein be arranged in two possible ways: parallel or serial [4,8-12]. In the serial structure, which has been the one applied in this study, the biological system dynamics are described by time differentials of classifying variables, while the unknown metabolic functions with intrinsic delays are handled by a nonparametric structure.

A general mathematical representation of delayed dynamics is given by Retarded Functional Differential Equations (RFDE) [13]. After applying certain simplifications, some special concepts arise such as models considering either discrete time delays, [14-16], distributed time delays, [1,17,18] or ordinary differential equations (ODE) of kinetic rates [2]. Although with varying performance, these models are shown to be capable of explaining the stability of biochemical networks [1,13,18-20].

Similar simplifications of RFDE as reported for parametric models can also be applied to hybrid semi-parametric models, i.e. either discrete delays or distributed delays of state variables in the kinetics or differential equations of the kinetics. The latter is not well suited for hybrid modelling, because neither kinetic function nor the kinetic values are known a priori and thus a solution or estimation of the kinetics is not straightforward. Distributed delays are also rather unlikely to be used, because one would have to introduce some function accounting for the delay, which is generally not known. Furthermore, some mathematical postulation of arbitrarily large delays for unknown weighting functions of the delayed variable would have to be assumed and this mathematical convenience is in limit biologically unrealistic (see [13]). Instead, the use of discrete delays in the inputs to the nonparametric structure is proposed herein. This is analogous to the application of discrete time series, namely **A**uto**R**egressive (e**X**ogenous), (AR(X)), models. This presents no limitation for application, as it is mathematically clear that a weighted discrete time series is equivalent to the integration of a time delay weighting function and thus analogous to the application of the distributed delay framework.

Unfortunately, the theoretically endless number of time lagged values of one variable would in practice lead to high computational times and identification problems of the network structure and parameters (see [21,22]). In theory, an optimal number of time lagged values exists given by the ratio between redundancy and additional gain of information in the inputs. Several methods for identification of the optimal number of time lagged values such as autocorrelation, cross-correlation, or partial mutual information, have been proposed [21,22]. However, they either assume that inputs are linearly correlated or are based on maximum information transfer and thus require known outputs, which unfortunately are not directly available in hybrid models.

Hence these methods cannot be applied here and thus the number of lagged values is rather chosen by trial and error, as done by several other authors [23,24]. However, choice by trial and error is not a disadvantage, when (i) the delay is an important property of the system and when (ii) series of delays are systematically studied, since it can be expected that models that account for time delays perform the best when the studied and the "true" delays are congruent.

In this paper hybrid delay differential equations with discrete time series was determined to be a powerful method to identify delayed dynamics of ill-defined biochemical networks. This technique is described in detail in the results section. The technique was applied to a typical gene regulatory system where the transport of macromolecules between the cytosol and nucleus introduce strong delay dynamic effects. In addition, heterologous protein expression by recombinant *Pichia pastoris *was studied by assuming a hypothetical network with distributed time delays.

## Results & Discussion

### Delay Differential Equation Hybrid Model (DDEHM)

Material balances over intracellular metabolites can be generically stated by the following dynamical equation

(1) $$\frac{dc_{int}}{dt}=K_{int}\cdot r_{int}+b_{int}-\mu \cdot C_{int}$$

where *c_int _*is a vector comprising the concentrations of intracellular metabolites, *K_int _*a *m *× *q *stoichiometric matrix of *m *metabolites and *q *metabolic reactions, *r_int _*a vector of *q *kinetic rates, *b_int _*a vector of transport fluxes across the cellular membrane and *μ *the specific growth rate.

If a macroscopic bioreactor model is formulated accounting only for the unbalanced extracellular metabolites, a similar equation is obtained which accounts for the volume dilution term (*D*·*c_ext _*) in substitution of the cell growth dilution term (*μ*·*c_int _*),

(2) $$\frac{dc_{ext}}{dt}=K_{ext}\cdot r_{ext}-D\cdot c_{ext}+u_{ext}.$$

Here *c_ext _*is a vector of concentrations of extracellular metabolites, *K_ext _*a matrix of stoichiometric coefficients, *D *is the dilution rate, *u_ext _*is a vector of volumetric feeding rates, and *r_ext _*is the kinetic rate vector.

All the results, presented from this point forward, are derived from eq. (2), which can however be automatically extended to eq. (1).

#### Delayed reaction kinetics

As suggested by [10], the vector of kinetic rates can be described either mechanistically, statistically or as a mixture of both types of models depending on the a priori knowledge about the metabolic network. A general definition is to state every metabolic flux as the multiplication of a mechanistic term (*ψ*) with an unknown nonparametric term (*ρ*) representing the unknown phenomena that must be identified from data.:

(3) r(X,w)=ψ(X)⋅ρ(X,w),

with *X *a vector of input variables and *w *a vector of empirical parameters. When no a priori mechanistic knowledge is available then the *ψ *term is dropped and eq. (3) reduces to

(4) r(X,w)=ρ(X,w).

As stated previously, the intrinsic causes of delays are the occurrence of several serial reaction steps with slow kinetics. To mimic this effect, and analogous to AR(X)models, both the *ψ *and *ρ *kinetic terms are modelled as a function of *X *, which includes discrete past values of metabolite concentrations, *c *,(that can be intracellular or extracellular, depending on the application of eqs. 1 or 2) and/or exogenous inputs:

(5) $$X=\left[\begin{array}{c} c_{i}(t),c_{i}(t-\tau_{i}),c_{i}(t-2\cdot \tau_{i}),...\;,c_{i}(t-N_{i}\cdot \tau_{i}),\\ s_{j}(t),s_{j}(t-\tau_{j}),s_{j}(t-2\cdot \tau_{j}),\;...\;,s_{j}(t-M_{j}\cdot \tau_{j}) \end{array}\right].$$

Here *c_i _*means value *i *of vector *c *, *τ_i _*is the associated time lag, *N_i _*defines the number of time lags assumed for each value *c_i _*of vector *c *, *s_j _*is the j^th ^exogenous input, *τ_j _*the associated time lag and its lag number is defined by *M_j _*. Note that the time lags and the numbers of time lags, *τ_i _*, *τ_j _*, *N_i _*, and *M_j _*can be chosen independently. However, it might be advantageous to model a time series around rough estimates of the "true" delays.

After considering eq. (2) - (5), it becomes clear that the model equations are Delay Differential Equations (DDE) in which the "retarded" or "lagged" phenomena are accounted by the reaction term, eq. (4).

Several linear or nonlinear regression methods can be used to formulate the unknown nonparametric kinetic function *ρ *. Here we adopted a three layer back propagation neural network with hyperbolic tangential activation function for the sake of comparability with other hybrid modelling studies since this method is the most reported in the literature [4,8-12]:

(6) $$\rho (X,w)=w_{2}\cdot g(w_{1}\cdot X+b_{1})+b_{2},$$

where *w *, the parameter vector, comprises the weights and biases, *w*_1 _, *w*_2 _, and *b*_1 _, *b*_2 _, respectively. The hyperbolic tangential activation function *g*(·) is,

(7) $$g(y)=\frac{1-e^{-2\cdot y}}{1+e^{-2\cdot y}}.$$

Note that the incorporation of AR into the hybrid approach results in delay differential equations, which is why the proposed hybrid model is referred to as the Delay Differential Equation Hybrid Model.

#### Nonparametric structure identification

The identification of the best network architecture by means of a trade-off between residual minimization, quantity of data and quantity of parameters is a central question when nonparametric models find application. This trade-off is due to the fact that more parameters on one hand will improve the fitting of the model to the data, but on the other hand might result in parameter over-fitting, leading to a degradation of model robustness or/and, even worse, in the addition of synthetic noise to the estimates [21,22].

The architecture of the Artificial Neural Network (ANN) structure involves the variation of the number of layers and the number of nodes. This variability is in this study, prior to application, already reduced by the selection of three layers, namely input, hidden and output layer. The application of three layers is usually sufficient if nonlinear continuous functions are sought to be modelled [22]. Remaining in terms of structural variability is such the evaluation of the variation of numbers of nodes for each hybrid model set-up.

#### Parameter identification

For each nonparametric structure, the respective parameters *w *must be estimated from data. In this paper a weighted least squares criteria of model residuals in concentrations is adopted:

(8) $$min\left\{E=\frac{1}{P\times n}\sum_{l=1}^{P}\sum_{i=1}^{n}\frac{{\left(c_{m,l,i}(t)-c_{l,i}(t,w)\right)}^{2}}{c_{\sigma ,i}}\right\},$$

where *P *is the number of samples, *n *is the number of state variables, *c_m, l, i _*are measured state variables, *c_l, i_*(*t*, *w*) are calculated state variables and *c_σ, i _*are the standard deviations. The serial hybrid structure, consisting of an ANN and material balances, was shown to be trained best by using the sensitivity approach along with analytical gradients [10]. Here we extended the sensitivity equations to the DDEHM case. The sensitivities equations are derived by differentiating eq. (2) with respect to *w *while taking into account the time lagged differential variables, which then reads as follows,

(9) $$\begin{array}{l} \frac{d}{dt}\cdot \frac{\partial c}{\partial w}=\;{\sum_{k=0}^{N_{i}}\left\{\frac{\partial (K\cdot \rho \cdot \psi )}{\partial c(t-k\cdot \tau )}\cdot \frac{\partial c(t-k\cdot \tau )}{\partial w}\right\}}_{\;\;}\\ \;\;\;\;\;\;\;\;\;\;\;\;\;\;\;\;\;\;\;\;+\frac{\partial K\cdot \psi \cdot \rho }{\partial w}-D\cdot I_{n}\cdot \frac{\partial c}{\partial w,} \end{array}$$

where

(10) $$\begin{array}{l} \sum_{k=0}^{N_{i}}\left\{\frac{\partial (K\cdot \rho \cdot \psi )}{\partial c(t-k\cdot \tau )}\cdot \;\frac{\partial c(t-k\cdot \tau )}{\partial w}\right\}=\\ \;\;\;\;\;K\cdot \rho \cdot \sum_{k=0}^{N_{i}}\left\{\frac{\partial \psi }{\partial c(t-k\cdot \tau )}\cdot \;\frac{\partial c(t-k\cdot \tau )}{\partial w}\right\}\;\;\\ \;+K\cdot \psi \cdot \sum_{k=0}^{N_{i}}\{\frac{\partial \rho }{\partial c(t-k\cdot \tau )}\cdot \frac{\partial c(t-k\cdot \tau )}{\partial w}\}, \end{array}$$

With *ρ *and *ψ *depending on the time lagged concentrations and where

(11) $$\frac{\partial K\cdot \psi \cdot \rho }{\partial w}=K\cdot \rho \cdot \frac{\partial \psi }{\partial w}+K\cdot \psi \cdot \frac{\partial \rho }{\partial w}\;;$$

For comparison of time-delay gradients for network training see [4,21].

This least square problem is solved by using the "lsqnonlin" Matlab function which uses a subspace trust region method and is based on the interior-reflective Newton method (Matlab Optimization toolbox) [25]. The sensitivity equations are integrated along with the delay differential model equations. This can either be accomplished using the dde23 Matlab algorithm, which integrates the delay differential equations with the explicit Runge-Kutta (2,3) pair and interpolant, or by using linear approximation of the differential equations for integration with storage of the respective delay values, which results in a time inexpensive algorithm. For the latter case, unfortunately, some error is introduced along with this simplification. However if average kinetic rates are estimated for each time step, the error is significantly diminished. Initial state values, *c*(*t*_0_), are problem dependent (for instance the initial concentration of biomass or substrate in a bioreactor). The initial values of the sensitivity equations are however zero $\left({\left(\partial c/\partial w\right)}_{t_{0}}=0,\;{\left(\partial c/\partial w\right)}_{t<t_{0}}=0\right)$, because the initial state values, *c*(*t*_0_), are independent of model parameters *w *. The residual gradients are then obtained using the corresponding sensitivity values. Notice that the lagged values of both state variables and exogenous inputs are assumed to be equal to the initial values *c*(*t*_0_) for all *t*-*N_i_*·*τ *<*t*_0 _.

Identification is initialized from a random selection of weight values as usually done for ANNs. The solution space is spanned by these weights and the identification, i.e. the objective to reduce the model residual, is a nonlinear optimization problem. Therefore, one cannot expect to obtain the global minimum as the result of the model's residuals minimum found from one random weight initialization. Instead, several iterations of the same set-up with random initialization should be carried out. The greater the number of random initializations, the greater the statistical confidence of the solution [21,22].

However, parameter identification is an iterative process which should be stopped when the model exhibits the best generalization of the target functions [21,22]. This is usually accomplished using two independent data sets: one for identification (also called training) which contains about 2/3 of all data points and another data set for validation with the remaining data. For these data sets some error criteria such as the Mean Least Square Error or the Bayesian Information Criterion (described in detail below) is calculated for the model residuals. Along the iterations, the best parameters are the ones where the selected criterion of the validation data set has its "best" value. A test data set can be used to additionally exploit the generalization capabilities.

#### Model performance criteria

The model residual, also addressed as the goodness of fit of the model estimates and the data, can be assessed with the Mean Square Error, MSE. The MSE decreases the better the fit and is defined as:

(12) $$MSE=\frac{1}{P\times n}\cdot {\sum_{I}\sum_{i}\left(c_{m,I,i}(t)-c_{I,i}(t,\;w)\right)}^{2}\cdot$$

This criterion is directly linked to the least square error which is used for parameter identification.

Due to the reason mentioned above, the MSE criterion is not addressed when it comes to architecture, structure, model comparison or selection. Appropriate criteria are (i) the Akaike Information Criteria, AIC, which is wildly used or (ii) the Bayesian Information Criteria, BIC, which is more appropriate for datasets with more than 46 data points [11,26,27]. Therefore the BIC is applied for model comparison and selection in this study. The BIC is defined as:

(13) $$\begin{array}{l} BIC=\left(-\frac{n\cdot P}{2}\cdot \ln\left(\sum_{I}\sum_{i}{\left[c_{m,I,i}\left(t\right)-c_{I,i}\left(t,w\right)\right]}^{2}\right)\right)\\ \;\;\;\;\;\;\;\;\;\;\;\;\;\;\;\;\;\;\;\;-\left(\frac{n_{W}}{2}\cdot \text{ln}\left(\frac{n\cdot P}{2\cdot \pi }\right)\right) \end{array}$$

where the term in the first bracket is the logarithmic maximum likelihood, *π *is the number "Pi" and *n_w _*is the total number of parameters/weights. In terms of the BIC, the model to be selected is the one that exhibits the larger BIC value for the validation set, see [11,26,27].

### Case Study I: Transcription Factor A (TF-A) dynamics with discrete time delay

**Figure 1:**
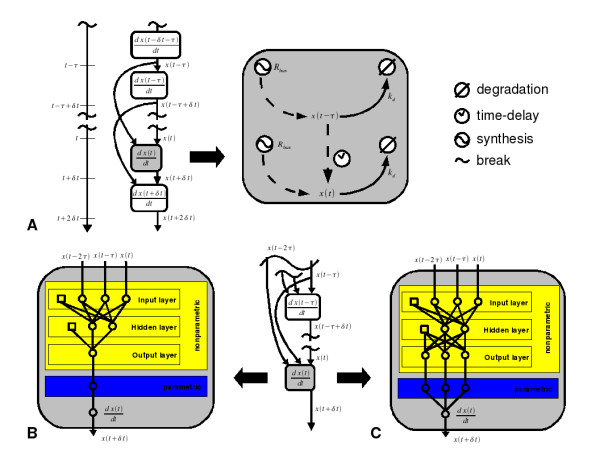
**Network Structures**. Delay TF-A transcription model. (A) true network structure (B) DDEHM network without prior knowledge, (C) DDEHM network with some prior knowledge. In structures (B) and (C), the ANN comprises three layers. The nodes of the input and output layer have linear transition functions, except for the input node of the time which has a hyperbolic tangential transition function as do the nodes of the hidden layer.

Genetic regulatory systems are built on signal transduction pathways through which specific transcription factors (TF) are phosphorylated. The phosphorylated TFs are then able to bind to responsive DNA sequences thereby regulating the transcription of nearby genes. Herein we consider the example of the TF-A model reported by [15] and [16] (see Fig. 1A). In this case, the TF activates its own transcription according to a typical positive feedback loop.

The translocation of macromolecules between cytosol and nucleus have a tremendous impact on gene regulation dynamics. Herein we consider a discrete delay for the translocation of TF-A as suggested by [15] and [16], giving rise to the following single delay differential equation describing the dynamics of the TF-A monomeric concentration in the nucleus, *x *:

(14) $$\frac{dx(t)}{dt}=\frac{\left(k_{f}\cdot x{\left(t-\tau \right)}^{2}\right)}{\left(x{\left(t-\tau \right)}^{2}+K_{d}\right)}x(t)\cdot K_{d}+R_{b\text{as}}$$

The first term on the right-hand side of eq. (14) is the rate of TF-A transcription in the cytosol which in the perspective of nucleus is affected by the translocation delay, *τ *=120 *min *. The second term refers to TF-A dissociation in the nucleus and the third term to a basal transcription rate, *R_bas _*, observed at very low TF-A concentrations.

**Figure 2:**
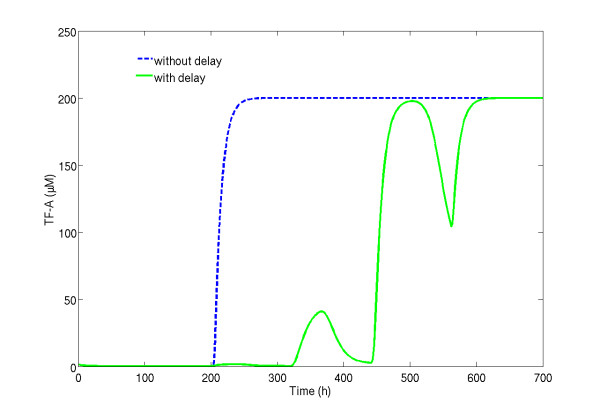
**Impact of delays on the TF-A profile**. Demonstration of the impact of the delay on the trajectory of TF-A transcription model over time. The TF-A model trajectory without delay is the blue dashed line while the TF-A trajectory with delay is the green continuous line.

Figure 2 shows the simulation of model eq. (14) with the parameters proposed by [15]. The TF-A dynamics are of a typical bistable system induced by the increase of the cytosol synthesis rate, *k_f _*, at time *t *= 200 *min *, forcing the system to jump to another state. The effect of the time delay can be assessed by comparing the full-line (with delay) with the dashed-line (without delay). The main consequence of the delay is that the TF-A concentration exhibits a "staircase" transition between the steadystates.

The main goal in this case study is to investigate if the TF-A delay dynamics, shown in Fig. 2, can be properly identified by the DDEHM framework proposed in this paper. With this goal in mind, 6 data sets of TF-A concentration in the nucleus over time with varying initial concentrations were generated (3 data sets with "clean" data, which were corrupted with white noise in order to obtain the training, validation and test set data).

#### Formulation and discrimination of a suitable DDEHM structure

The two DDEHM structures, shown in Fig. 1B and 1C, were identified from the simulation data. In the former structure, no prior knowledge about the TF-A network is incorporated while in the latter case some prior knowledge inspired in eq. (14) and in autoregulated systems is considered.

In preliminary studies, we concluded that structure (1c) leads to both a faster convergence and improved results than structure (1b) (results not shown). This observation is in line with the study reported by [10], where it was shown that including a priori knowledge in the hybrid structure generally improves their identification capacity.

A selection of results obtained with structure (1c) are presented in Table 1 showing model performance criteria for the training, validating and testing data sets (namely MSE and BIC) over structure parameters. Overall, it can be observed that structures without time delays are in general outperformed by those containing time delays if one of the effectual delays is close to the "true" delay, i.e. a model with a delay mismatch as high as 10% still gives an improved performance in comparison to no delay at all (Table 1). It can also be noticed that the MSE values for the case of one delay tend to improve the closer the effectual delay gets to the "true" delay, peaking when the effectual is the true delay. Also, it strikes that the best models (highest BIC values for the validation set) are obtained mostly for 4-7 nodes in the hidden layer, an observation that reflects the complexity of the addressed system. Owed to this complexity, are also the strayed deviations in the overall consistent performance in terms of BIC. The consideration of series of delays also gives rise to consistent models, especially if only two delays are considered. When three delays are considered, model performance increases with decreasing number of nodes, which contrasts with the results obtained for one or two delays. Even so, the best values therewith are obtained with 4 numbers of nodes. While the good model performances are due to the fact that the "true" delay is present in the applied models, the slightly worse performance when compared to the single delay models sources from the evitable, additional information. Evitable (correlated) information hampers the model structure identification [21,22], which explains why the model performance for three considered delays decreases with the increasing number of nodes.

**Table 1:** Results for Case Study I

NN	τ*_i_*		BIC			MSE		NN	τ*_i_*		BIC			MSE	
					
		train	valid	test	train	valid	test			train	valid	test	train	valid	test
5	0	-12217	-5836	-5997	0.0141	0.0152	0.0210	6	0	-12220	-5869	-6039	0.0139	0.0157	0.0222

2	100	-13118	-6209	-6190	0.0368	0.0350	0.0337	2	110	-13058	-6150	-6157	0.0347	0.0310	0.0315

3	100	-13087	-6273	-6336	0.0350	0.0384	0.0437	3	110	-13043	-6269	-6275	0.0334	0.0381	0.0385

4	100	-11826	-5650	-5888	0.0096	0.0105	0.0170	4	110	-12273	-5805	-5832	0.0151	0.0144	0.0152

5	100	-11386	-5379	-5733	0.0060	0.0059	0.0120	5	110	-12302	-5864	-6008	0.0152	0.0156	0.0210

6	100	-12873	-6174	-6176	0.0265	0.0282	0.0284	6	110	-13162	-6336	-6329	0.0355	0.0392	0.0386

7	100	-13144	-6269	-6176	0.0342	0.0330	0.0273	7	110	-11516	-5572	-5731	0.0066	0.0081	0.0111

2	120	-13047	-6148	-6139	0.0343	0.0309	0.0303	2	130	-13242	-6332	-6371	0.0417	0.0449	0.0486

3	120	-12105	-5782	-5960	0.0130	0.0142	0.0204	3	130	-13076	-6173	-6203	0.0346	0.0314	0.0333

4	120	-11974	-5761	-5891	0.0111	0.0132	0.0171	4	130	-12652	-6087	-6090	0.0221	0.0254	0.0256

5	120	-11436	-5462	-5489	0.0062	0.0068	0.0071	5	130	-11823	-5604	-5676	0.0094	0.0092	0.0107

6	120	-10820	-5170	-5714	0.0033	0.0036	0.0108	6	130	-12679	-6093	-6108	0.0218	0.0240	0.0247

7	120	-12533	-6002	-5881	0.0184	0.0193	0.0151	7	130	-13269	-6384	-6393	0.0388	0.0417	0.0424

2	140	-13069	-6155	-6167	0.0351	0.0313	0.0321	2	160	-13195	-6295	-6257	0.0398	0.0416	0.0385

3	140	-12303	-5805	-5803	0.0158	0.0149	0.0149	3	160	-12252	-5823	-5771	0.0151	0.0155	0.0139

4	140	-13288	-6375	-6384	0.0420	0.0455	0.0464	4	160	-13063	-6186	-6241	0.0334	0.0311	0.0347

5	140	-12537	-6043	-6039	0.0193	0.0225	0.0223	5	160	-12022	-5716	-5909	0.0114	0.0116	0.0171

6	140	-12564	-6067	-6078	0.0194	0.0228	0.0233	6	160	-12052	-5800	-5995	0.0116	0.0133	0.0197

7	140	-11439	-5535	-5994	0.0061	0.0075	0.0189	7	160	-11466	-5431	-5441	0.0063	0.0061	0.0062

2	80, 120	-13016	-6146	-6079	0.0330	0.0305	0.0266	2	120, 160	-12984	-6137	-6027	0.0320	0.0299	0.0240

3	80, 120	-12334	-5860	-5968	0.0162	0.0164	0.0204	3	120, 160	-13115	-6296	-6163	0.0357	0.0397	0.0303

4	80, 120	-11221	-5276	-5566	0.0051	0.0048	0.0087	4	120, 160	-12250	-5872	-5934	0.0145	0.0162	0.0183

5	80, 120	-12780	-6221	-6207	0.0243	0.0314	0.0305	5	120, 160	-12293	-5872	-5984	0.0148	0.0155	0.0194

6	80, 120	-12233	-5837	-5944	0.0136	0.0139	0.0172	6	120, 160	-11240	-5352	-7991	0.0050	0.0052	1.0762

7	80, 120	-11688	-5663	-5630	0.0077	0.0094	0.0088	7	120, 160	-11703	-5623	-6004	0.0078	0.0086	0.0187

2	80, 120,160	-12994	-6144	-6034	0.0321	0.0300	0.0241	5	80, 120, 160	-12487	-5953	-6045	0.0178	0.0178	0.0215

3	80, 120, 160	-11855	-5641	-5937	0.0099	0.0104	0.0189	6	80, 120, 160	-12824	-6193	-6213	0.0244	0.0276	0.0288

4	80, 120, 160	-11879	-5605	-5734	0.0099	0.0092	0.0120	7	80, 120, 160	-12167	-5758	-5774	0.0122	0.0110	0.0113

Effect of structure parameters (number of nodes in the hidden layer, NN, and number and values of time delays) on the performance of the structure displayed in Fig. 1C. For every structure incorporating delays two random initial weight sets were investigated. For those without delays four different random initial weight changes were investigated. At least 25 iterations were carried out for each set of weights. The number of iterations was expanded if network learning was observed during the last iterations. Integration of the material balances along with the differential equations resulting from the sensitivity method for parameter identification is carried out for this simulation case with the dde23 MATLAB function for the studies with delays, and with the ode23 MATLAB function for the ones without delays. This results in higher simulation times, but as the dimension of the set of equations is rather small, the total simulation time is maintainable.

The most consistent structure with highest predictive power has 5 nodes in the hidden layer and a single delay coincident to the "true" delay of 120 min. The respective BIC value was -5489 while the MSE value was 0,0071 for the test data set. The best structure without time delays, which had also 5 nodes in the hidden layer, showed a fourfold increase in the MSE value for the test data set (0,0210) and a considerably lower BIC value (-5997). This result clearly demonstrates the advantage of the delay hybrid modelling approach proposed in this paper.

#### Comparison of best structures with and without time delays

**Figure 3:**
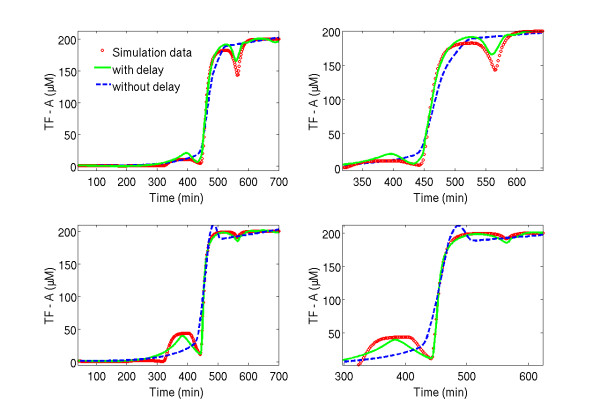
**Qualitative results on the time curse of TF-A**. TF-A modelling results for the two runs of test data. On the left side the whole simulation region of the data set is shown while on the right side the most interesting section of the respective data set is highlighted: red circles are "measured" TF-A data over time, green line are the identification results by model structure (1C) with 5 hidden nodes and *τ *=120 minutes; blue dashed line are the identification results by model (1C) with 5 hidden nodes and no time delay.

Figure 3 compares the modelling results for the two best structures with delay *τ *=120 *min *and without delay for the validation and test data sets. It can be seen that the model without time delay provides a very smooth transition between the two steady-states. However, the "true" dynamics, i.e. the staircase transition function, of the measured data are not met. In contrast, the model with *τ *=120 *min *was able to capture these "staircase" dynamics. The curves both increase slightly in the beginning until a time value of about 320 min, where the first "stair" appears in the data.

Thereafter, a significant increase can be noticed until a time of about 390 min, where the estimate has a first maximum peak. Then the concentration estimation decreases until a time point of about 450 min. Subsequently, in both cases, the data points are almost completely met by the estimates in the time interval from 450 until 650 min.

### Case Study II: Heterologous protein expression by MUT+ *Pichia pastoris*

In methanol utilizing MUT+ *Pichia pastoris *strains, fast phenotype, foreign protein expression is controlled by the promoter of the alcohol oxidase gene 1 (AOX1). In typical culture conditions, the yeast cells are first grown on glycerol to reach a certain optimal cell density. Glycerol and most carbon sources other than methanol strongly repress AOX1, thus product is not formed in this phase. Then methanol feeding induces AOX1 over 1000-fold [28] thereby initiating foreign protein expression. The transition between glycerol and methanol phases can take between 1 to 4 hours depending on the strategy for methanol feeding. This transition phase corresponds to the time needed by the cells to express the alcohol oxidase enzyme, which is an essential enzyme for the cells to metabolize methanol. Apart from this delay in the transition phase, time delays between methanol uptake, biomass growth and product formation were also observed during the post-induction phase, [2]. In the paper by Bellgardt and co-workers [2], a so called extended regulator model was adopted which is somewhat analogous to a linear distributed time delay kernel of the specific protein synthesis rate over the specific growth rate. The inclusion of such a delay model was essential to fit their data, although the underlying biological fundamentals are not clearly understood. The effects causing such time delays seem to be a principal part of the *Pichia pastoris *systems. However, they are poorly studied [29] and mechanistically not understood. Thus the nature of the apparent delays can mechanistically not be precisely defined (i.e. as a discrete delay model), wherefore a distributed delay model is the most appropriate representation.

**Figure 4:**
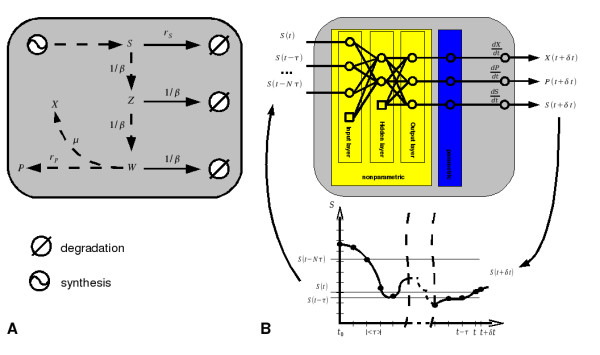
***Pichia pastoris *network with delay dynamics**. (A) network with a quadratic distributed time delay kernel of cell growth and protein expression over methanol uptake. The respective equations are listed in Table 2. This network was used to generate simulation data (B) Approximation of network (A) by a hybrid network. Structure (B) was investigated to see if the novel framework is able to identify unknown distributed delay dynamics.

**Figure 5:**
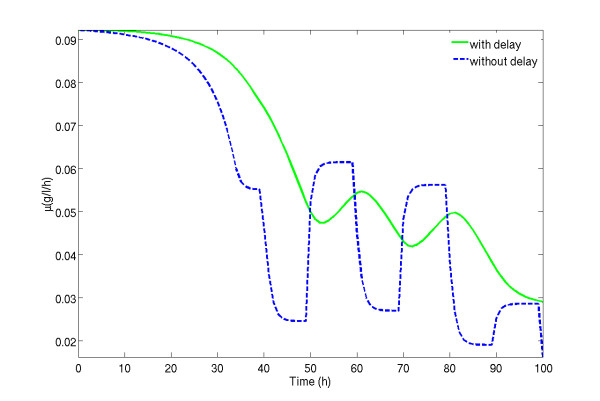
**Impact of delays on the specific biomass growth rate**. Green full line, is the specific growth rate when considering the network shown in Fig. 4A; blue dashed line is the specific growth rate when no delay between substrate uptake and biomass growth is considered.

The main goal in this section is to determine if the hybrid methodology proposed herein is able to effectively identify such unknown distributed time delay dynamics in *P. pastoris*. The *P. pastoris *network shown in Fig. 4A was used as a case study. This network includes a quadratic distributed delay kernel (Eqs. A5 and A6 of Table 2), which is considered as a strong delay kernel, see Fig. 5. In this case, the cell growth rate and the foreign protein expression rate are delayed in relation to the methanol uptake. The corresponding model equations are listed in Table 2. Note that the reactor balance equations are also listed since they are an important element to generate consistent simulation data.

**Table 2:** Mathematical model for data generation

Reactor model equations:	
(A1) $\frac{dX(t)}{dt}=X(t)\cdot \mu (W(t))+D\cdot X(t)$	(A2) $\frac{dS(t)}{dt}=-r_{\text{s}}(S(t))\cdot X(t)-D\cdot (S(t)-S_{F})$

(A3) $\frac{dP(t)}{dt}=r_{\rho }(W(t))\cdot X(t)-D\cdot P(t)$	(A4) $\frac{dV(t)}{dt}=F(t)$

(A5) $\frac{dW}{dt}=\frac{Z-W}{\beta }$	(A6) $\frac{dZ}{dt}=\frac{S(t)-Z}{\beta }$

(A7) $F(t)=\left(\frac{V(t)}{S_{F}-S(t)}\right)\cdot \left(r_{\text{s}}\cdot X(t)+\frac{S_{set}-S(t)}{\tau_{set}}\right)$	(A8) $D=\frac{F}{V}$

**Cell model equations:**	

(A9) $\mu =K_{B1}\cdot \frac{W(t)}{K_{s}+W(t)}-K_{B2}\cdot m_{ATP}$	(A10) $r_{\rho }=K_{\rho 1}\cdot \mu +K_{\rho 2}$

(A11) $r_{S}=r_{S,max}\cdot \frac{S(t)}{K_{s}+S(t)}$	(A12) $W(t)=\int_{-\infty }^{t}(S(t-\tau )/\beta^{2}).\;\tau \cdot \exp(-\tau /\beta )\cdot d\tau$

Parameters and initial Values:

*D *,-, (1/*h*); *F *,-, (*g*/*l*); *S_set _*,10, (*g*/*l*); *K_B1 _*,0.1184, (1/*h*); *K_B2 _*,4.7376, (*g*/*mol*); *K_P1 _*,0.48, (-); *K_P2 _*,0.0008, (1/*h*); *K_s _*,10, (*g*/*l*); *m_ATP _*,0.0015, (*mol*/(*g.h*)); *P *,0, (*mg*/*l*); *r_s, max _*,0.19, (1/*h*); *S *,40, (*g*/*l*); *S_F _*,1260, (*g*/*l*); *t *,-, *h *; *V *,15, *l *; *W *, *W*_0 _= *S*_0 _, (*g*/*l*); *X *,1, (*g*/*l*); *Z *, *Z*_0 _= *S*_0 _, (*g*/*l*); *τ_set _*,1, *h *; *β*, *h*; *μ *,-, (1/*h*);

Mathematical model of MUT+ *Pichia pastoris *expression with a quadratic distributed delay kernel. This model was used to generate six data sets. Three of which contain the clean, noise-free data and the other three the associated white noise corrupted data. One data set of the noise corrupted sets was used to train the hybrid model, one was used for validation and the third one for testing. Integration was performed with the ode45 MATLAB function which integrates the differential equation with a Runge-Kutta (4,5) integration scheme. The obtained state variables, namely concentrations of biomass, substrate and product, the reactor volume and as well the feed concentration are recorded and assumed as measured data for the evaluation. Variation in the data was obtained by application of varying initial values, i.e. the initial values were 5% Gaussian distributed. Note that model equations (A5 and A6) are derived from equation (A 12) using the linear chain trick [17,18] and that (A 12) is never used for model calculations.

#### Identification of distributed time delays by DDEHM

The hybrid model structure shown in Fig. 4B was adopted to identify the network, Fig. 4A. The neural network assumes no prior knowledge about Fig. 4B and uses as the external excitation signal present delayed methanol concentration values. The specific methanol uptake rate, *r_S_*(*t*), specific growth rate, *μ*(*t*), and specific product synthesis rate, *r_P_*(*t*), are the target kinetic variables that need to be identified. Note that in the real system *μ*(*t*) and *r_P_*(*t*) are delayed in relation to *r_S_*(*t*) according to a quadratic distributed delay kernel (see Eqs. A9-A12 in Table 2). These three kinetic rates are passed to the macroscopic reactor material balances for the calculation of the respective concentrations.

Table 3 shows model performance parameters (BIC and MSE) for the hybrid model structure (Fig. 4B) with varying number of hidden nodes (between 2 and 8) and different series of lagged input variables (between 0 and 4 with intervals of 2 or 2.5 hours). In general, the BIC values for the two series of lagged variables are much better than when no lagged variables are considered. This again confirms the advantages of the DDEHM methodology proposed herein for identifying delayed dynamics.

**Table 3:** Results for Case Study II

NN	Nlag	*τ*	BIC			MSE			NN	Nlag	*τ*	BIC			MSE		
							
			train	valid	test	train	valid	test				train	valid	test	train	valid	test
3	0	0	-18561	-5147	-5000	0.0155	0.0187	0.0157	6	0	0	-18500	-5199	-5030	0.0148	0.0187	0.0154

4	0	0	-18514	-5164	-4994	0.0155	0.0187	0.0150	7	0	0	-18576	-5197	-4990	0.0153	0.0180	0.0144

5	0	0	-18531	-5186	-5011	0.0151	0.0189	0.0153	8	0	0	-18430	-5211	-5053	0.0146	0.0182	0.0158

2	1	2	-17144	-4697	-4475	0.0086	0.0098	0.0065	2	1	2.5	-16343	-4293	-4331	0.0077	0.0076	0.0067

3	1	2	-16981	-4588	-4470	0.0085	0.0108	0.0067	3	1	2.5	-17230	-4725	-4584	0.0186	0.0327	0.0183

4	1	2	-16877	-4581	-4484	0.0087	0.0117	0.0072	4	1	2.5	-16635	-4506	-4690	0.0154	0.0220	0.0170

5	1	2	-16792	-4544	-4490	0.0078	0.0093	0.0064	5	1	2.5	-16927	-4614	-4524	0.0086	0.0111	0.0072

6	1	2	-16911	-4643	-4544	0.0087	0.0119	0.0073	6	1	2.5	-16944	-4523	-4488	0.0082	0.0092	0.0066

7	1	2	-17197	-4632	-4505	0.0097	0.0151	0.0084	7	1	2.5	-17044	-4742	-4608	0.0082	0.0107	0.0075

8	1	2	-17181	-4530	-4496	0.0123	0.0155	0.0105	8	1	2.5	-16976	-4694	-4608	0.0084	0.0110	0.0076

2	2	2	-16466	-4512	-4525	0.0067	0.0079	0.0067	2	2	2.5	-17813	-4833	-4759	0.0130	0.0152	0.0129

3	2	2	-16856	-4656	-4535	0.0081	0.0116	0.0073	3	2	2.5	-16637	-4684	-4595	0.0079	0.0123	0.0079

4	2	2	-16788	-4616	-4525	0.0079	0.0111	0.0072	4	2	2.5	-16703	-4507	-4517	0.0073	0.0083	0.0064

5	2	2	-16734	-4430	-4446	0.0075	0.0088	0.0061	5	2	2.5	-16384	-4327	-4404	0.0068	0.0074	0.0063

6	2	2	-16573	-4271	-4353	0.0077	0.0081	0.0065	6	2	2.5	-16601	-4400	-4432	0.0071	0.0079	0.0062

7	2	2	-16704	-4569	-4541	0.0071	0.0089	0.0065	7	2	2.5	-16569	-4405	-4466	0.0068	0.0072	0.0061

8	2	2	-16921	-4728	-4632	0.0082	0.0132	0.0079	8	2	2.5	-16833	-4790	-4664	0.0080	0.0127	0.0079

2	3	2	-19006	-5136	-5080	0.0181	0.0177	0.0158	2	3	2.5	-16619	-4566	-4514	0.0077	0.0099	0.0071

3	3	2	-16811	-4692	-4549	0.0078	0.0119	0.0073	3	3	2.5	-16037	-4218	-4259	0.0064	0.0072	0.0058

4	3	2	-16737	-4474	-4466	0.0069	0.0089	0.0063	4	3	2.5	-16439	-4224	-4287	0.0068	0.0078	0.0057

5	3	2	-16519	-4357	-4408	0.0066	0.0076	0.0058	5	3	2.5	-16199	-4358	-4295	0.0063	0.0076	0.0056

6	3	2	-16832	-4506	-4415	0.0090	0.0107	0.0078	6	3	2.5	-16604	-4577	-4556	0.0072	0.0094	0.0067

7	3	2	-16565	-4385	-4439	0.0066	0.0072	0.0058	7	3	2.5	-16344	-4475	-4432	0.0064	0.0078	0.0060

8	3	2	-16758	-4672	-4569	0.0079	0.0117	0.0069	8	3	2.5	-16471	-4374	-4505	0.0066	0.0069	0.0061

2	4	2	-16655	-4365	-4504	0.0071	0.0107	0.0077	2	4	2.5	-16562	-4532	-4503	0.0079	0.0097	0.0073

3	4	2	-16377	-4301	-4431	0.0067	0.0078	0.0064	3	4	2.5	-16325	-4471	-4470	0.0072	0.0086	0.0066

4	4	2	-16215	-4183	-4316	0.0062	0.0067	0.0057	4	4	2.5	-16261	-4189	-4281	0.0064	0.0068	0.0058

5	4	2	-16611	-4481	-4484	0.0070	0.0101	0.0066	5	4	2.5	-15954	-4190	-4255	0.0056	0.0060	0.0052

6	4	2	-17503	-4762	-4597	0.0189	0.0374	0.0197	6	4	2.5	-16439	-4334	-4476	0.0064	0.0073	0.0060

7	4	2	-25835	-7171	-7226	10.160	9.2368	11.843	7	4	2.5	-25949	-7288	-7280	13.781	19.040	17.824

8	4	2	-16256	-4328	-4369	0.0058	0.0061	0.0052	8	4	2.5	-16296	-4326	-4381	0.0061	0.0065	0.0054

Results of the performance measures, BIC and MSE, over structure parameters, namely Numbers of Nodes in the hidden layer of the ANN, NN, Number of time lags, Nlag and the time lag, τ, for *Pichia pastoris *cells with distributed time delays using the structure of Fig. 4B. Integration of material balances along with the equations obtained from the sensitivity method is carried out using the linear approximation integration schema described in the Methods section. The times series were chosen such that one of the delays matched the maximum of the time delay of the weighting function of the simulation case (see Eqs. A 12).

#### The effect of discrete time delays

It can be further noticed that BIC values of the time series with a time delay of 2.5(*h*) are slightly better when compared to the ones for time series with a 2(*h*) time delay. This observation agrees with the results of the previous case study where the performance of the hybrid model peaked the closer the model delay was to the true delay. In this case the maximal weighted delay is 5(*h*). Moreover, the BIC values tend to improve with increasing number of lagged variables. The increasing number of input lagged variables, which are weighted by the neural network, seem to result in more accurate discrete time approximations of the continuous distributed time delays. In contrast, it can also be observed in Table 3 that with increasing number of delays the best BIC value is more likely to be found for a lower number of nodes in the hidden layer of the ANN. However, this was expected as the BIC is constrained by the number of model parameters. Nevertheless, the same observation is made for the MSE values. Furthermore, it was observed that significantly more random changes of the parameter values were required when the numbers of incorporated delays increased in order to achieve results which were coherent with the ones obtained for smaller numbers of delays.

#### Comparing standard and DDE hybrid models

**Figure 6:**
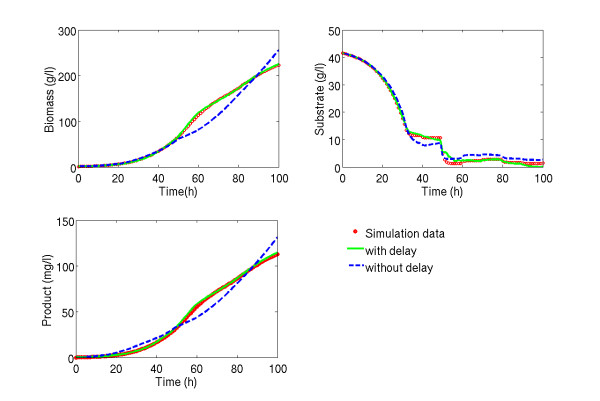
**Qualitative results for trajectories of concentrations**. *Pichia Pastoris *distributed delay modelling result for a fed-batch of the test data set: red circles are "measured" data over time; green line are the identification results by model structure (4B) with 5 hidden nodes and a series of 4 time lagged variables of 2.5 hours; blue dashed line are the identification results by model (4B) with 7 hidden nodes and no time delay.

Figure 6 shows non-noisy simulation data and the best modelling results of hybrid models with and without delays for the concentrations of biomass, substrate and product in a fed-batch of the test set. In the figures of biomass and product concentrations, predictions of the hybrid models with delays are practically congruent to the true process behaviour. The intrinsic dynamics of the organism are perfectly met. In clear contrast are the predictions of the hybrid standard model without time delays. Biomass and product concentrations are under-predicted for a time span between 50 to 85 h, then followed by over-prediction from 85 h till the end. Before 50 h only insignificant differences between predictions and data are visible. For the substrate concentration, the DDEHM model shows a significant amount of error in a short time window from about 48 to 55 h, which is coincident to a fast decrease in the substrate concentration. It should be noticed that such fast dynamics are rather challenging to integrate (see comments below). As for the standard hybrid model without delays, it predicts accurately substrate dynamics only at the beginning, i.e. from 0 to 30 h. Thereafter, between 30 and 50 h, the model under-predicts substrate concentration, and after 50 h, it over-predicts the substrate concentration.

#### Note about numerical integration of DDEs

The integration of the hybrid model differential equations using the built-in MATLAB solvers (dde23, ode23) showed to be computationally intensive. A typical training run took 4 to 5 days. Moreover, convergence was sometimes not accomplished due to the limitation of the integration step size and accuracy. On the other hand, the integration of the hybrid model differential equations with the linear approximation only lead to small discrepancies for substrate concentrations if the step size was chosen adequately small, i.e. between 0.05-0.1 h. Convergence was tested by decreasing even further the integration step without significant changes in the integration results. This approach lead to a reduction of computation of about 75% (i.e. 3 days) when compared to the MATLAB solvers.

## Conclusions

Time-delays have a profound impact on cellular regulatory mechanisms. Therefore, their modelling is essential in metabolic engineering and process optimization studies. The detailed mechanisms behind observed time delays are often unknown. The required "omic" data for a fundamental mathematical modelling of such phenomena is generally unavailable at the required time resolution and accuracy. As a result, biochemical delayed dynamics are many times only "measurable" through their external consequences in terms of extracellular properties. We propose herein a hybrid semi-parametric modelling method to identify such delayed dynamics. The principle is probing from outside to understand the inner workings. The concept was applied to two illustrative case studies. The overall results show that significantly better prediction qualities of the novel hybrid model when compared to the traditional approach were obtained in all case studies, being the more distinctive the more significant the underlying system delay is. When system and model delay are identical the model quality peaked but even with a delay mismatch as high as 10% in the TF-A gene-regulatory network, modelling results were significantly enhanced in comparison to no delay at all. These results support a system delay identification strategy by studies of different discrete delays in the input variables. For the studies on *Pichia pastoris *with intrinsic distributed time delays significant enhancements were introduced by the DDEHM model. This suggests that even though the proposed structure bases on discrete time delays directly of external excitation variables, it poses no limitation of applicability. In conclusion the method proposed herein is a powerful tool to identify time delays in ill-defined biochemical networks.

## Authors' contributions

The project was conceived by RO, JP and SFA. The algorithms were developed by RO and MVS. Data analysis was performed by MVS and RO and manuscript writing were performed by MVS, JP, SFA and RO. All authors read and approved the final manuscript.
